# Liberal Offers

**Published:** 1878-10-20

**Authors:** 


### LIBERAL OFFERS.

New subscribers for the year 1879 may commence with the forthcoming November or December numbers.

Any one sending us two new subscribers and $4 for 1879 will be furnished a copy of Leonard's Physician's Day Book; or Lindsay & Blakeston's Visiting List for 1879.

